# The Commercialisation of Medical and Scientific Reporting

**DOI:** 10.1371/journal.pmed.0010038

**Published:** 2004-12-28

**Authors:** Timothy Caulfield

## Abstract

Commercial influences on research results can in turn lead to "hype" in science and medicine news stories

There is a growing recognition of the importance of the popular press in the communication of science. The media is often the primary source of science information and, as such, can have a profound impact on how the public views the risks and benefits of scientific advances. Dorothy Nelkin suggests that the “media serve as brokers between science and the public, framing the social reality for their readers and shaping the public consciousness about science related events” [1].

Because of this influential role, many commentators have been highly critical of the quality of media reporting, suggesting that reporting is “hyped”, irresponsible, and hurtful to the public's understanding of important scientific issues. In 1999, the United Kingdom House of Commons Science and Technology Committee was concerned enough to recommend that the media be governed by a Code of Practice that “stipulates that scientific stories should be factually accurate” [2].

But is it fair to point an accusing finger solely at the popular press? There are many examples of science reporting that has been less than perfect, such as the coverage of behavioural genetics and human cloning. And there is no doubt that an entertaining or controversial spin will win out over a muted message. But there is also evidence that the media does a surprisingly good job [3], often accurately conveying information found in peer-reviewed journals. A more subtle problem, and one that may have more long-term implications than simply bad reporting, is the *faithful* portrayal of commercially influenced research results.

## A Marketing Message?

There is an expanding body of evidence that suggests that the increasingly commercial nature of biomedical research is having an impact on how science stories are portrayed. Studies have shown that papers in peer-reviewed journals are more likely to contain positive findings if the research is funded by industry [4]. A study that examined pharmaceutical research found that “among the authors of original research papers, reviews and letters to the editor that were supportive of the drugs' use, 96% had financial relationships with the drugs' manufacturers; for publications deemed neutral or critical the figure was only 60% and 37% respectively” [5,6]. To make matters worse, there is also evidence that negative results are either de-emphasised or simply not published [7,8]. This bias is picked up by the popular press and conveyed, largely uncritically, to the public [3].[Fig pmed-0010038-g001]

**Figure pmed-0010038-g001:**
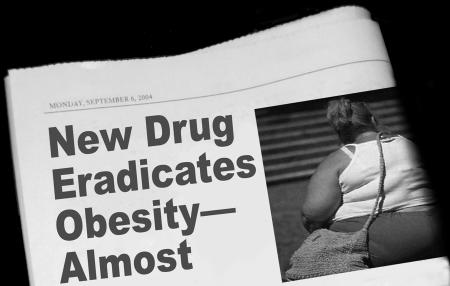
Commercial influences can spin a story

Commercial influence on public representations of science has the potential to create a skewed picture of biomedical research—a picture that emphasises benefits over risks, and predictions of unrealistic breakthroughs over a tempered explanation of the incremental nature of the advancement of scientific knowledge. In the area of genetics, for example, there is concern that this commercial influence will lead to a simplistic and overly deterministic view of the role of genes in human health and may have an adverse impact on public dialogue [1]. There is also concern that it will create unrealistic expectations about a given scientific advance or product. In the context of health care, this may lead to inappropriate and expensive utilisation patterns.

Given the increasingly close connection between the media's portrayal of science and the broader agenda of commercialisation, some media representations can be viewed as a subtle form of marketing, albeit often inadvertent. One commentator has gone so far as to suggest that, to a large degree, “medical news is actually unpaid advertising” [9].[Boxed-text box1]

**Further Reading on Media Hype**

BlumDKnudsonM1997A field guide to science writing: The official guide of the national association of science writersNew YorkOxford University Press304pBernhardtBAGellerGTamborEMountcastle-ShahEMullJGet al2000Analysis of media reports on the discovery of breast and prostrate cancer susceptibilityAm J Hum GenetSuppl 27662CaulfieldT2000Underwhelmed: Hyperbole, regulatory policy and the genetic revolutionMcGill Law J4543746012688281CohnVCopeL2001News and numbers: A guide to reporting statistical claims and controversies in health and related fields, 2nd edAmes (Iowa)Iowa State University Press211pConditCM1998Determinism and mass media portrayals of geneticsAm J Hum Genet62979984952934210.1086/301784PMC1377024ConradP1999Uses of expertise: Sources, quotes, and voice in the reporting of genetics in the newsPublic Underst Sci8285302GarfieldE1979SIPI: Scientists taking scientific information to the publicCurr Contents41290293GellerGBernhardtBHoltzmanN2002The media and public reaction to genetic researchJAMA28777311851549MoynihanRBeroCLRoss-DegnanDHenryDLeeKet al2000Coverage by the news media of the benefits and risks of medicationsN Engl J Med342164516501083321110.1056/NEJM200006013422206NelkinDLindeeMS1995The DNA mystique: The gene as a cultural iconNew YorkW. H. Freeman312pHargreavesILewisJSpeersT2003Toward a better map: Science, the public and the mediaSwindon (United Kingdom)Economic and Social Research CouncilAvailable: http://www.esrc.ac.uk/esrccontent/DownloadDocs/Mapdocfinal.pdf. Accessed 28 September 2004RansohoffDRansohoffR2001Sensationalism in the media: When scientists and journalists may be complicit collaboratorsEff Clin Pract418518811525108

This is not to say that science reporting is part of a coordinated effort to promote a particular product. On the contrary, there is rarely a specific product to promote, and the media is just looking for an interesting and intriguing story that will help sell papers. However, in the long run, a continued, systemic trend toward positive, industry-influenced reporting may operate in much the same way as an explicit promotional campaign. In fact, optimistic media portrayals could be considered more powerful than promotional campaigns. The message is separated from an obvious marketing agenda and often includes a trusted voice, such as a university-based researcher. Paradoxically, this trust is based in part on a belief in the perceived independence of university researchers.

## Balancing the Message

There is nothing inherently wrong with commercial involvement in biomedical research. After all, in most countries with an advanced biomedical research infrastructure, commercial entities, rightly or not, are an essential element of the technology-development process. Nevertheless, we need to be sensitive to the influence of market forces on how science is represented to the public. Eventually, the public will catch on. And when they do, public trust in the biomedical research enterprise may be irreparably harmed.

In an increasingly knowledge-based economy, there seems to be little doubt that private industry will continue to play a significant role in the funding of science. The research community must adjust to this inevitability by taking steps to ensure that portrayals of science remain as balanced as possible. As thoughtfully noted by Tom Wilkie: “If science is to manage the transition from its older, academic tradition to a new style, while keeping popular assent and the popular image of science as an impartial means of getting at the truth, then the scientific community itself must recognise the importance of maintaining impartial sources of public information.” [10]

What can be done? For a start, reporters should always ask for and researchers should always offer information about the nature of the funding and the financial relationship of the researchers to the sponsor. Increasingly, this information is disclosed in peer-reviewed journals. However, it may not be communicated in other popular representations of research results. As motivation, the research community should remember that the media also likes a good conflict-of-interest story [11]. Complete transparency should be the understood standard practice.

The research community should also consider the establishment of various sources of independent science information, including a venue for the publishing of negative results and a list of respected researchers who may be able to provide an alternative view. Not only would this create an outlet for results that do not correspond with commercial interests, it would also serve as a resource for the media. Reporters are often under extremely tight deadlines, and it is not always easy to find an independent second opinion, an indispensable component of balanced reporting.

Naturally, commercial pressure isn't the only source of science hype, and it is understandable that researchers may want to promote their latest findings. But commercial influence is emerging as a known source of bias, and it is a phenomenon that could have a profound impact on how the public perceives the entire research enterprise. Developing strategies, starting with the modest ones suggested above, seems to be an essential element of any communication strategy.
